# The association between ambient air pollution and birth defects in five major ethnic groups in Liuzhou, China

**DOI:** 10.1186/s12887-021-02687-z

**Published:** 2021-05-14

**Authors:** Xiaoli Huang, Jichang Chen, Dingyuan Zeng, Zhong Lin, Carly Herbert, Lesley Cottrell, Liu Liu, Arlene Ash, Bo Wang

**Affiliations:** 1 Liuzhou Maternity and Child Healthcare Hospital, 50 Yingshan Street, Liuzhou, 545003 Guangxi Zhuang Autonomous Region China; 2 Department of Population and Quantitative Health Sciences, Albert Sherman Center, University of Massachusetts Medical School, 368 Plantation Street, Worcester, MA 01605 USA; 3 Department of Pediatrics, West Virginia University School of Medicine, Morgantown, WV. One Medical Drive, Morgantown, WV 26506 USA

**Keywords:** Ambient air pollution, Birth defects, Ethnic groups, Liuzhou

## Abstract

**Background:**

Studies suggest that exposure to ambient air pollution during pregnancy may be associated with increased risks of birth defects (BDs), but conclusions have been inconsistent. This study describes the ethnic distribution of major BDs and examines the relationship between air pollution and BDs among different ethnic groups in Liuzhou city, China.

**Methods:**

Surveillance data of infants born in 114 registered hospitals in Liuzhou in 2019 were analyzed to determine the epidemiology of BDs across five major ethnic groups.

Concentrations of six air pollutants (PM2.5, PM10, SO2, CO, NO2, O3) were obtained from the Liuzhou Environmental Protection Bureau. Logistic regression was used to examine the associations between ambient air pollution exposure and risk of BDs.

**Results:**

Among 32,549 infants, 635 infants had BDs, yielding a prevalence of 19.5 per 1000 perinatal infants. Dong ethnic group had the highest prevalence of BDs (2.59%), followed by Yao (2.57%), Miao (2.35%), Zhuang (2.07%), and Han (1.75%). Relative to the Han ethnic group, infants from Zhuang, Miao, Yao and Dong groups had lower risks of congenital heart disease, polydactyly, and hypospadias. The Zhuang ethnic group had higher risks of severe thalassemia, cleft lip and/or palate, and syndactyls. Overall BDs were positively correlated with air pollutants PM_10_ (aOR =1.14, 95% CI:1.12 ~ 2.43; aOR =1.51, 95% CI:1.13 ~ 2.03 for per 10μg/mg3 increment) and CO (aOR =1.36, 95% CI:1.14 ~ 2.48; aOR =1.75, 95% CI:1.02 ~ 3.61 for every 1 mg /m3 increment) in second and third month of pregnancy. SO_2_ was also significantly associated with BDs in the second month before the pregnancy (aOR = 1.31; 95% CI: 1.20 ~ 3.22) and third month of pregnancy (aOR =1.75; 95% CI:1.02 ~ 3.61). Congenital heart disease, polydactyl, cleft lip and/or palate were also significantly associated with PM_10_, SO_2_ and CO exposures. However, no significant association was found between birth defects and O_3_, PM_2.5_ and NO_2_ exposures (*P* > 0.05).

**Conclusion:**

This study provides a comprehensive description of ethnic differences in BDs in Southwest China and broadens the evidence of the association between air pollution exposure during gestation and BDs.

**Supplementary Information:**

The online version contains supplementary material available at 10.1186/s12887-021-02687-z.

## Background

Birth defects (BDs) are functional, structural, or metabolic abnormalities that occur before birth, usually as a result of chromosomal abnormalities, congenital malformations, genetic and metabolic disorders, and functional abnormalities [1]. They are an important cause of abortion, stillbirth, and death of pregnant mothers, as well as the leading cause of infant and child death in the first year of life [2, 3]. The most common major structural BDs include congenital heart disease, neural tube defects, lip cleft, limb reduction defect, and Down syndrome [4]. Studies have shown that BDs account for approximately 2.3 to 3% of total live births [5].

China is the most populous country in the world, with one of the highest incidences of documented BDs. In China, the estimated prevalence of BDs is approximately 4 to 6% [1]. In recent years, with the rapid development of urbanization and the rise of industrial modernization, air pollution has become an increasingly serious threat to human health.

Numerous studies have shown that ambient air pollutants have a direct negative impact on the birth outcomes of pregnant women [6, 7]. Exposure to ambient air pollutants during pregnancy can lead to preterm delivery, fetal growth restriction, and other adverse pregnancy outcomes such as BDs [8, 9].

However, studies on the relationship between ambient air pollution and BDs are inconsistent, and the timing of BD susceptibility during pregnancy as a result of ambient air pollution is unclear. A study in the United States showed that exposure to NO2 in early pregnancy increases the risk of congenital abnormalities in newborns [10]. Additional studies have shown that exposure to a certain concentration of NO2 before or during the first trimester increases the incidence of birth defects [11]. Wang et al. conducted a time series study on the impact of air pollution on birth defects in Xi’an city, China, in which atmospheric pollutants SO2, NO2 and PM10 were shown to have an impact on BDs [12]. In a previous study conducted in Lanzhou, exposure to PM_10_ during the whole pregnancy, early pregnancy and middle pregnancy was correlated with the occurrence of patent ductus arteriosus, a congenital heart malformation. However, the previous study did not consider the influence of ethnic factors on BDs [13]. A case control study of pregnant women in Fuzhou from 2007 to 2013 also showed a positive correlation between PM_10_ exposure and fetal cardiovascular malformation [14]. Strickland et al. observed a significant association between PM_10_ and patent ductus arteriosus during weeks three to seven of pregnancy [15]. In a population-based case-control study in Hunan province from 2014 to 2016, they found that SO_2_ had a greater effect on the prophase of pregnancy, while PM_10_ had an effect in late third trimester [16]. Vrijheid et al. concluded that SO_2_ exposure was related to coarctation of the aorta and tetralogy of fallot, two congenital heart defects [17]. However, other studies have reported that PM_10_ and SO_2_ have no association with BDs [18]. Some studies have found insignificant effects of ambient air pollution on BDs, and some pollutants have even demonstrated protective effects [8]. Further, few studies have examined the impact of ambient air pollution prior to conception as a risk for BDs. Differences in study results may be due to differences in region, race, study design, covariate control, exposure assessment, statistical methods, and sample size. Compared with the global literature on BDs and air pollution, China has relatively less evidence on this topic; further, research on ambient air pollution in China has been mainly concentrated in economically developed areas in eastern China, such as Beijing, Tianjin, Hebei [19, 20], Yangtze River Delta [21, 22] and the Pearl River Delta [23]. There are few studies in the western region of China, specifically within ethnic minority areas.

Liuzhou is located in southwestern China and is the main gathering place for ethnic minorities in Guangxi province, China. Ethnic minorities account for 37.2% of the population of Guangxi Province [24]. By the end of 2018, the population of Liuzhou City was 4.04 million. The ethnic composition of Liuzhou residents has reached more than 30 ethnic groups. Among the permanent residents of Liuzhou, the Han ethnic group has the largest population (48.9%), followed by the Zhuang (35.2%), Miao (6.4%), Dong (6.3%), Yao (1.9%), and Molao (0.8%) [24–26]. The Han ethnicity is the largest population in China, accounting for approximately 91.5% [24]. The second largest ethnicity is Zhuang, accounting for approximately 1.3% [24]. Ethnic and racial differences in the prevalence of BDs have been described in the United States, with results showing a lower risk of BDs among African Americans and Hispanics populations compared with Caucasians and Asians [27]. Additionally, in the United States, racial and ethnic differences in BD risk were associated with cultural, social experience or genetic susceptibility [28, 29]. It is possible that ethnic groups in China may show similar differences in BD risk. Liuzhou is an important industrial town in Guangxi, and the problem of ambient air pollution is becoming increasingly serious, with the regional natural ecological environment extremely fragile. The incidence of chronic diseases, maternal mortality, and low birth weight among minority ethnic groups in China are also high, making this population worthy of attention [30, 31].

Using the ambient air pollution monitoring network and the BDs monitoring system, we investigated all infants born in ethnic minority areas of Liuzhou between January 2019 and December 2019 to study whether air pollution was associated with an increased risk of BDs. We also calculated the mean daily gestational concentrations of environmental pollutants during 3 months before pregnancy and the first trimester of pregnancy. This study also examines differences in the prevalence of BDs among five major ethnic groups and provides a reference for the prevention of birth defects in minority areas.

## Methods

### Data resources

Research data were extracted from the birth defect monitoring sub-module in the Liuzhou Maternal and Child Health Information Management System between January 2019 and December 2019. This includes records from perinatal babies, including live birth, stillbirth and infant death within 7 days, reported by 114 midwifery agencies in Liuzhou for a total of 32,549 births. The data excluded twin and multiple births. Among this cohort, there were 635 cases of BDs. All data was derived under the supervision of the health administration, and this study was approved by the Institutional Review Board of Liuzhou Maternal and Child Health Hospital.

Additional perinatal and maternal data were derived from the China Maternal and Child Health Monitoring Data Direct Reporting System (https://zhibao3.mchscn.org/) and the Maternal and Child Health System of Liuzhou City. Using the “Birth Defects Registration Card” and “Quarterly Report on Number of Perinatal Births” from the Maternal and Child Health System of Liuzhou City, relevant data were collected, including maternal status, birth status, birth defect diagnosis and family history. Maternal data included ethnicity, age, education, family income, date of last menstrual period, residential address, registration address and parity. Infant data included date of birth, sex, gestational age, fetal number, weight, and outcome (including stillbirth, fetal death or live birth between 20 weeks of gestation through 7 days after birth).

Family history included abnormal fertility history and family genetic history. The diagnosis of BDs was based on the “International Statistical Classification of Diseases and Related Health Problems, Tenth Edition” (ICD-10) and Chinese National Criteria of BDs [32]. We investigated a wide range of birth defects including 11 major types of BDs: hydrocephaly, congenital heart disease, cleft lip with or without cleft palate, urinary system abnormalities, ear anomalies, congenital clubfoot, polydactyly and congenital syndactyly, severe thalassemia, Cystic hygroma, and Bart’s Syndrome. Any birth defects not included in these 11 diagnoses were categorized as “other”.

### Quality controls

In order to ensure the accuracy of the report, a physician at each registered hospital was required to complete a quarterly form, in addition to the Birth Defects Registration Card. Each quarterly table contained 3 months of data, including ethnicity, date of last menstrual period, parity, education, family income, date of birth, gestational age, weight, number of births, whether labor was inducted after diagnosis of a birth defect, diagnostic basis, diagnosis of malformation, and birth defect diagnosis for each birth occurring in the hospital. Birth defect registration cards and quarterly tables were reviewed and audited by maternal and child health hospitals and health administrative departments. Quality control measures were monitored regularly in respective hospitals, quarterly at the county level and every two years at the municipal or provincial level. The quality requirements for BDs monitoring data included: 100% completion rate of form, form items error rate less than 1%, input error rate less than 1%, and a rate of missed birth defects less than 1%.

### Exposure assessment

**Fig. 1:**
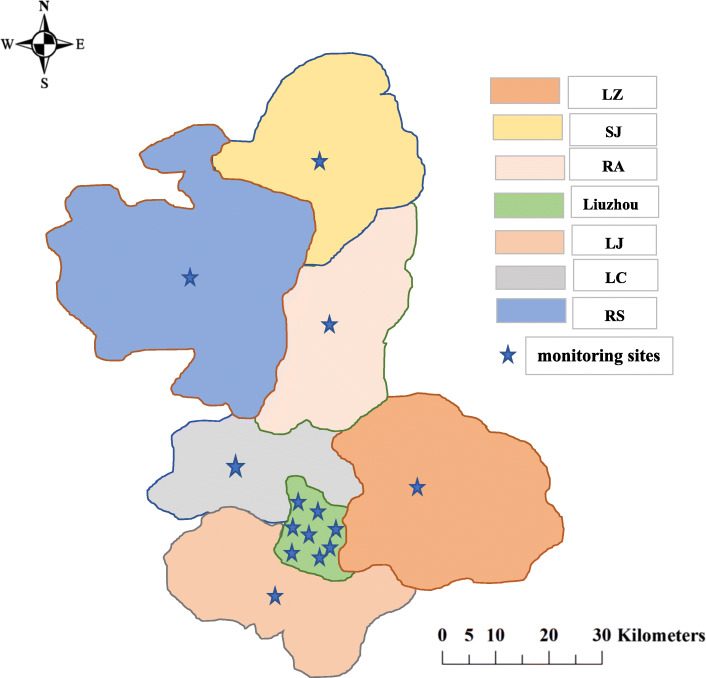
Location of study area (8 districts) and air quality monitoring sites in Liuzhou

The ambient air pollution data used in this study came from the weather information data collected by the Liuzhou Environmental Protection Bureau between January 2018 and December 2019, which includes six state-controlled air automatic monitoring points in Liuzhou (HX Waterworks, Liuzhou Fourth Middle School, GTS, Environmental Monitoring Station, Liudong Primary School, Liuzhou Ninth Middle School), two district control stations (Liuzhou Second middle school, LW), and six city and county control stations (LJ District Experimental High School, LC County Middle School, LZ County Youth Activity Center, RA County Quality Supervision Bureau, RS County Health School, the SJ County Guyi Town Center) (Fig. 1). Monitored pollutants included particulate matter less than 2.5 μm in aerodynamic diameter (PM2.5), particulate matter less than 10 μm in aerodynamic diameter (PM10), sulfur dioxide (SO2), Carbon monoxide (CO), Nitrogen dioxide (NO2), and ozone (O3). O3 was calculated as a maximum daily average over 8 h. The daily concentration values of other pollutants were calculated based on the average of 24 h measured at 14 monitoring points.

The assessment was performed according to the national standard “Ambient Air Quality Standard” (GB 3095–2012). The average annual secondary concentration limits of PM2.5, PM10, SO2 and NO2 are 35 μg/m^3^, 70 μg/m^3^, 60 μg/m^3^ and 40 μg/m^3^, respectively. The average 24-h secondary concentration limits of PM2.5, PM10, SO2, CO, NO2 and O_3_ are 75 μg/m^3^, 150 μg/m^3^, 75 μg/m^3^, 4 mg/m^3^, 80 μg/m^3^, and 160 μg/m^3^, respectively.

Maternal ambient air exposure was determined through block kriging, a statistical technique which predicts average exposure concentrations based on spatial variation. The daily concentrations of PM_2.5_, PM_10_, SO_2_, CO, NO_2_ and O_3_ on the dates between last menstrual period and date of delivery were determined based on maternal residential address. Monthly concentrations for the 3 months prior to pregnancy and the first trimester of pregnancy were estimated for each participant [33, 34].

### Covariates

Potential covariates considered from the birth defects registration cards included maternal age (< 20 years, 20–24 years, 25–29 years, 30–34 years, ≥35 years), family income (<2000RMB, 2000–3999 RMB, 4000–7999 RMB, ≥8000 RMB), highest education levels (classified as primary school or below, middle school, high school/technical school, college or above), birth weights (< 1500 g, 1500 g–2499 g, 2500 g–3499 g, ≥3500 g), number of pregnancies (1, 2, and ≥ 3), total previous live births (0, 1, and ≥ 2), and gender of infant (male, female). We also adjusted for additional maternal covariates according to the existing literature and the study population characteristics. We collected variables suspected as potential confounders from the China Maternal and Child Health Monitoring Data Direct Reporting System. These potential confounders included premature rupture of membrane, syphilis, ethnicity, hyperthyroidism, gestational diabetes, preeclampsia, gestational hypertension, HIV, hypothyroidism, infection, medication and in vitro fertilization-embryo transfer. Maternal smoking and alcohol use during pregnancy were not controlled for because < 0.3% of the mothers reported smoking or drinking alcohol.

### Statistical methods

Chi-square test was performed to examine the differences in social demographics between infants with BDs and infants without BDs. Prevalence rates of overall BDs and each specific BD were also examined. Distributions of air pollutant concentrations were presented by quartile and interquartile range (IQR) averaged over three months preconception and during the first trimester of pregnancy. Logistic regression was used to assess the association of air pollution exposure on BDs. BDs were the dependent variable, and the individual exposure concentration of air pollutants during three months prior to pregnancy and the first trimester of pregnancy were the independent variables. Important covariates were controlled, in order to explore the influence of exposure concentration and exposure time on BDs. To assess the role of ambient air pollutant exposure at different stages of pregnancy, we constructed exposure variables for the 3 months before pregnancy and the first trimester of pregnancy. We studied both the impact of a single pollutant on BDs and the combined effects of multiple air pollutants. Corresponding odds ratio (OR) and 95% confidence interval (95% CI) were calculated for birth defects and air pollutant exposure at different stages of pregnancy. In order to evaluate the robustness of the estimated effects, we performed sensitivity analyses measuring the associations between ambient air pollution and birth defects in Han and Zhuang ethnic groups (sample sizes for other ethnic groups are relatively small). Statistical test significance level is 0.05 (two-tailed). Data processing and statistical analysis were performed using SAS 9.4 (SAS Institute Inc., Cary, NC, USA) statistical software package.

## Results

### Distribution of selected characteristics in the study population

Table 1 shows the characteristics of the study population. Of the 32,549 perinatal infants included in analysis, 635 had BDs, for a prevalence of 19.50 per 1000 infants. There were statistically significant differences between infants with and without BDs in terms of maternal age, maternal education, birth weight, infant gender, total previous live births, and residence (*p* < 0.01). There were no statistically significant differences between infants with and without BDs for family income and number of pregnancies (*p* > 0.05).

**Table 1:** Characteristics of subjects in Liuzhou city, Guangxi Province

Characteristics	Infants with BDs n (%)	Normal infants n (%)	_χ_2	P
Maternal age (years)				
< 20	19(3.0%)	936 (2.9%)	11.10	< 0.01
20 ~	73 (11.5%)	4271 (13.4%)		
25 ~	191 (30.1%)	10,359 (32.5%)		
30 ~	199 (31.3%)	10,517 (33.0%)		
≥ 35	153 (24.1%)	5831 (18.3%)		
Family income (RMB per capita)				
< 2000	156 (24.6%)	7662 (23.5%)	2.54	0.16
2000~	107 (16.8%)	5087 (15.6%)		
4000~	250 (39.4%)	13,147 (40.4%)		
≥ 8000~	123 (19.4%)	6653 (20.4%)		
Maternal education				
Primary or below	28 (4.4%)	128 (0.4%)	9.49	0.00
Middle school	184 (29.0%)	11,567 (35.5%)		
High or technical school	124 (19.5%)	10,543 (32.4%)		
College or above	299 (47.1%)	10,311 (31.7%)		
Birth weight(g)				
< 1500 g	316 (49.8%)	194 (0.6%)	2723.66	< 0.01
1500 g ~	52 (8.2%)	1799 (5.6%)		
2500 g ~	201 (31.6%)	22,424 (70.3%)		
≥ 3500 g	66 (10.4%)	7497 (23.5%)		
Infant gender				
Male	361 (56.8%)	16,790 (52.6%)	14.50	< 0.01
Female	233 (36.7%)	15,124 (47.4%)		
Unknown gender	41 (6.5%)	0 (0.0%)		
Number of pregnancies				
1	110 (17.3%)	6591 (20.7%)	1.30	0.25
2	175 (27.6%)	9130 (28.6%)		
≥ 3	350 (55.1%)	16,193 (50.7%)		
Total previous live births				
0	97 (15.3%)	21 (0.1%)	740.78	< 0.01
1	304 (47.9%)	12,259 (38.4%)		
≥ 2	234 (36.8%)	19,634 (61.5%)		
Residence				
Urban	250 (39.4%)	15,510 (48.6%)	15.50	< 0.01
Rural	385 (60.6%)	16,404 (51.4%)		

### Description of BDs in the five major ethnic groups

Table 2 shows the rate of BDs among the five major ethnic groups in Liuzhou city. The Dong ethnic group had the highest prevalence of total BDs (2.59%), followed by Yao (2.57%), Miao (2.35%), Zhuang (2.07%), and Han (1.75%).

**Table 2:** The prevalence of BDs in the five major ethnic groups in Liuzhou city, China from January 1, 2019 to December 31, 2019

Ethnic	Number of birth defect(n)	Prevalence of Birth defects (%)	Number of perinatal infants(n)
Han	278	1.75	15,907
Zhuang	244	2.07	11,799
Dong	49	2.59	1890
Miao	45	2.35	1918
Yao	13	2.57	505
Others	6	1.15	530

### Description of BD types in the five major ethnic groups

BD diagnoses in the five major ethnic groups is presented in Table 3. The top five classes of BDs were congenital heart disease, polydactyly, cleft lip and/or cleft palate, severe thalassemia, and malformations of the external ear. The top five birth defect classes comprised over 59% of the 635 birth defects in total. There were 163 cases of congenital heart disease, comprising 25.67% of all birth defects analyzed. Relative to the Han ethnic group, Zhuang, Miao, Yao and Dong groups had a lower risk of congenital heart disease, polydactyly and hypospadias. Zhuang had higher risk of severe thalassemia, cleft lip and/or cleft palate, and syndactyls.

**Table 3:** Top ten classes of total BDs and ethnic distribution in Liuzhou

Birth defects	Case (n)	Incidence (%)	Han n (%)	Zhuang n (%)	Dong n (%)	Miao n (%)	Yao n (%)
Congenital heart disease	163	25.67	70 (11.02)	63 (9.92)	14 (2.20)	11 (1.73)	4 (0.63)
Polydactyly	70	11.02	31 (4.88)	22 (3.46)	5 (0.79)	8 (1.26)	1 (0.16)
Cleft lip and/or cleft palate	67	10.55	19 (2.99)	32 (5.04)	4 (0.94)	5 (0.79)	2 (0.31)
Severe thalassemia	40	6.30	13 (2.05)	19 (2.99)	1 (0.16)	5 (0.79)	1 (1.59)
Ear anomalies	38	5.98	20 (3.15)	15 (2.36)	2 (0.31)	1 (0.16)	0
Hypospadias	25	3.94	12 (1.89)	8 (1.26)	4 (0.63)	1 (0.16)	0
Congenital syndactyly	24	3.78	8 (1.26)	10 (1.57)	2 (0.31)	4 (0.63)	0
Congenital clubfoot	22	3.46	10 (1.57)	7 (1.10)	3 (0.47)	2 (0.31)	0
Hydrocephaly	21	3.31	11 (1.73)	6 (0.94)	1 (0.16)	2 (0.31)	1 (0.16)
Cystic hygroma	20	3.15	10 (1.57)	7 (1.10)	2 (0.31)	1 (0.16)	0
Bart’s Syndrome	13	2.05	6 (0.94)	5 (0.70)	1 (0.16)	1 (0.16)	0
Other Birth Defects	42	6.61	30 (4.72)	10 (1.57)	0	1 (0.16)	1 (0.16)

### Average ambient air pollution exposure level at different gestational time points

Individual ambient air pollution exposure levels at different gestational time points are shown in Table 4. The level of individual exposures (mean ± SD) to PM2.5 was highest (43.18 ± 19.46) three months prior to pregnancy. O3 exposures were highest (83.77 ± 11.69) two months prior to pregnancy, PM10 and NO2 exposures were highest (65.90 ± 37.05 and 27.97 ± 16.69) in first month of pregnancy, and SO2 and CO were highest (24.86 ± 23.00 and 1.18 ± 0.29 respectively) in the third month of pregnancy.

**Table 4:** Summary statistics for each pollutant during different gestation in Liuzhou, China

	Mean ± SD	Minimum	Maximum	25th PCTL	50th PCTL	75th PCTL
Before pregnancy						
1st month						
PM2.5 (μg/m^3^)	41.95 ± 16.03	15.35	89.78	29.45	40.31	53.35
PM10 (μg/m^3^)	64.97 ± 31.93	26.49	218.56	44.36	58.36	77.33
O3 (μg/m^3^)	80.91 ± 16.30	17.78	108.78	74.28	83.28	91.22
SO2 (μg/m^3^)	20.74 ± 19.17	4.92	218.87	14.13	19.39	23.35
NO2 (μg/m^3^)	27.01 ± 14.23	10.56	102.32	19.38	22.16	32.35
CO (mg/m^3^)	1.06 ± 0.25	0.63	1.92	0.91	1.14	1.21
2nd month						
PM2.5	41.45 ± 15.72	14.98	131.32	28.48	39.75	50.02
PM10	61.66 ± 23.30	25.78	217.46	43.46	57.93	77.11
O3	83.77 ± 11.69	16.54	109.21	77.25	86.37	92.22
SO2	18.72 ± 11.19	3.45	217.89	14.13	19.32	22.32
NO2	25.27 ± 9.86	9.43	101.73	19.28	22.27	30.03
CO	1.02 ± 0.20	0.58	1.87	0.96	1.11	1.21
3rd month						
PM2.5	43.18 ± 19.46	4.67	118.45	27.65	40.18	57.34
PM10~	61.50 ± 21.40	31.32	218.46	42.87	58.42	77.36
O3	82.03 ± 14.00	17.46	108.32	75.43	83.43	92.03
SO2	18.78 ± 8.44	4.32	218.68	13.94	18.27	22.35
NO2	24.71 ± 8.00	10.46	102.98	18.75	22.17	30.43
CO	1.03 ± 0.21	0.67	1.96	0.89	1.02	1.23
**Pregnancy**						
1st month						
PM2.5	41.78 ± 16.93	15.67	88.67	29	39.36	53.37
PM10	65.90 ± 37.05	25.67	217.95	43	50.43	56.45
O3	76.30 ± 21.03	17.79	107.74	68	81.37	91.76
SO2	22.23 ± 23.34	4.46	217.58	15	19.03	24.36
NO2	27.97 ± 16.69	9.08	101.67	18	22.17	32.42
CO	1.11 ± 0.28	0.64	1.87	0.9	1.10	1.31
2nd month						
PM2.5	39.78 ± 17.25	15.56	89.63	27	35.44	52.38
PM10	63.18 ± 38.45	25.66	217.63	42	49.38	72.17
O3	72.06 ± 22.83	17.12	108.58	51	78.47	90.04
SO2	23.20 ± 23.48	4.12	218.57	15	20.11	25.17
NO2	27.98 ± 17.30	9.09	102.37	18	22.03	32.47
CO	1.14 ± 0.30	0.68	1.92	0.9	1.12	1.33
3rd month						
PM2.5	38.25 ± 16.94	6.06	89.89	27	35.48	46.34
PM10	62.88 ± 38.27	25.45	218.47	42	50.43	72.46
O3	68.30 ± 23.65	17.59	107.74	46	74.47	89.52
SO2	24.86 ± 23.00	4.67	217.83	16	21.22	28.49
NO2	27.67 ± 17.37	7.09	101.87	18	22.36	33.27
CO	1.18 ± 0.29	0.69	1.96	1.0	1.22	1.37

*Abbreviations*: *SD* standard deviation, *1st month* The first month exposure, *2nd month* The second month exposure, *3rd month* The third month exposure, *PCTL* percentile

### Crude and adjusted odds ratios for BDs associated with air pollutants during different gestational time points

Table 5 shows the crude and adjusted odd ratios for BDs in relation to air pollutants at different gestational time points, spanning from three months prior to pregnancy through the first trimester of pregnancy. We observed a significant association between BDs and PM10 particularly in second (aOR =1.14; 95% CI:1.12–2.43) and third months of pregnancy (aOR = 1.51; 95%CI:1.13–2.03). SO2 had also a significant association with BDs for every 10 μg /m^3^ increase in concentration during two months prior to pregnancy (aOR =1.31; 95%CI:1.20–3.22) and the third month of pregnancy (aOR =1.75; 95%CI:1.02–3.61). CO also had a significant association with BDs for every 1 mg /m^3^ increase in concentration in the second (aOR =1.36; 95%CI:1.14–2.48) and third months of pregnancy (aOR =1.75; 95%CI:1.02–3.61). However, no significant association was found between birth defects and O3, PM2.5 and NO2 (*P* > 0.05). We observed similar associations in sensitivity analyses in Han and Zhuang, ethnic groups, respectively.

**Table 5:** Crude and adjusted odd ratios for BDs associated with air pollutants during different gestational periods

			Crude			Adjusted	
		OR	95%CI	P	OR	95%CI	P
PM2.5	Before pregnancy						
	1st month	1.02	0.99–1.00	0.34	1.00	1.00–1.01	0.53
	2nd month	0.93	0.89–0.98	0.76	1.05	0.96–1.15	0.27
	3rd month	1.00	0.97–1.26	0.35	0.99	0.94–1.13	0.32
	Pregnancy						
	1st month	1.00	0.99–1.03	0.46	1.07	0.96–1.15	0.26
	2nd month	0.97	0.86–1.01	0.47	1.15	0.99–1.21	0.16
	3rd month	0.83	0.75–0.98	0.91	1.05	0.98–1.16	0.27
PM10	Before pregnancy						
	1st month	1.00	0.99–1.07	0.84	1.01	0.98–1.05	0.78
	2nd month	1.01	1.00–2.03	0.00	1.00	0.99–1.07	0.76
	3rd month	1.31	1.04–3.21	0.00	1.12	1.03–2.01	0.22
	Pregnancy						
	1st month	1.06	1.00–1.20	0.03	1.00	0.94–1.31	0.06
	2nd month	1.73	1.60–2.00	0.01	**1.14**	**1.12–2.43**	**0.02**
	3rd month	1.61	1.44–2.10	0.07	**1.51**	**1.13–2.03**	**0.02**
O3	Before pregnancy						
	1st month	0.93	0.87–0.99	0.00	0.94	0.89–1.03	0.62
	2nd month	0.96	0.95–0.98	0.00	1.02	0.93–1.12	0.17
	3rd month	0.98	0.97–1.18	0.00	0.96	0.88–1.06	0.45
	Pregnancy						
	1st month	0.89	0.87–0.99	0.02	0.83	0.99–1.00	0.74
	2nd month	0.99	0.96–1.07	0.00	1.00	0.99–1.05	0.84
	3rd month	1.00	0.99–1.12	1.01	1.00	0.99–1.06	0.45
SO2	Before pregnancy						
	1st month	1.03	1.00–1.21	0.00	1.00	1.00–1.06	0.58
	2nd month	3.01	2.01–4.07	0.00	**1.31**	**1.19–3.22**	**0.04**
	3rd month	1.01	0.99–1.06	0.00	0.94	0.89–1.01	0.27
	Pregnancy						
	1st month	1.00	0.94–1.06	0.01	1.01	1.00–1.06	0.16
	2nd month	0.96	0.87–1.02	0.86	0.98	0.82–1.01	0.36
	3rd month	1.99	1.99–3.98	0.00	**1.75**	**1.02–3.07**	**0.02**
NO2	Before pregnancy						
	1st month	1.00	0.92–1.01	0.53	1.00	0.89–1.03	0.55
	2nd month	1.02	1.01–1.08	0.00	1.02	1.00–1.42-	0.13
	3rd month	1.03	1.02–1.14	0.00	1.01	1.00–1.32	0.39
	Pregnancy						
	1st month	1.00	0.99–1.21	0.94	1.01	1.00–1.07	0.14
	2nd month	1.27	1.04–1.75	0.40	0.89	0.84–1.01	0.13
	3rd month	1.99	1.39–2.03	0.03	0.91	0.88–1.11	0.72
CO	Before pregnancy						
	1st month ~	2.70	2.03–3.59	0.00	1.29	0.92–1.83	0.14
	2nd month	5.35	3.67–7.80	0.00	1.42	0.93–2.17	0.10
	3rd month	2.11	1.46–3.04	0.00	1.03	0.67–1.59	0.89
	Pregnancy						
	1st month	1.90	1.47–2.47	0.00	1.24	0.90–1.71	0.19
	2nd month	1.26	0.97–1.63	0.08	**1.36**	**1.14–2.48**	**0.03**
	3rd month	0.92	0.70–1.21	0.55	**1.75**	**1.02–3.61**	**0.02**

*Abbreviations*: *OR* odd ratio, *CI* confidence interval. Models were adjusted for maternal age, maternal education, birth weight, infant gender, total previous live births, residence and other air pollutants within the same exposure period

Congenital heart disease, polydactyl, cleft lip and/or palate were significantly associated with PM_10_, SO_2_ and CO. The sensitive period of these three birth defects caused by air pollution is consistent with that of total birth defects (supplemental Tables 1, 2 and 3).

## Discussion

In this population-based study, we utilized Liuzhou City’s perinatal birth defect monitoring and survey data to describe the ethnic distribution of major BDs, examine the difference in prevalence rate of BDs among major ethnic groups, and evaluate the correlation between maternal exposure to ambient air pollution and BDs in Liuzhou, Guangxi, China. The prevalence of total BDs among perinatal infants was 19.53 per 1000 births in 2019, which is lower than the prevalence rate of 25.2 per 1000 births reported in Liuzhou in 2011–2016 [35]. This study uniquely analyzed all pregnant outcomes from midwifery institutions and community health services, including live birth, stillbirth and infant death within 7 days, expanding the scope of the research to include those giving birth outside the hospital setting.

Although the overall prevalence rate of BDs was lower than before, the prevalence rate among ethnic minorities exhibited an upward trend in Liuzhou. We found that the Dong ethnic group had the highest prevalence of total BDs, followed by Yao, Miao, Zhuang and Han ethnic groups. Our result was inconsistent with previous studies in Liuzhou, which showed that minorities were less likely to have BDs than the Han ethnic group [35]. In our study, the upward trend in the epidemiology of BDs among ethnic groups may be explained by several factors. One reason may be attributed to the advancement of medical technology in China, which has improved prenatal diagnoses and screening techniques in ethnic minority areas and aided in timely and effective diagnosis of BDs. The Chinese government has also established an online BDs monitoring system nationwide to avoid missed reporting, increasing the accuracy of the data overtime.

In this study, the top five BDs reported were congenital heart disease, polydactyly, cleft lip and/or cleft palate, severe thalassemia, and malformations of the external ear. The incidence of congenital heart disease was the highest, which was consistent with the monitoring results of most hospitals [36, 37]. Relative to the Han ethnic group, Zhuang had higher risk of severe thalassemia, cleft lip and/or cleft palate and syndactyls. The etiology of BDs are complex and include multiple risk factors, such as advanced age, teratogenic exposures during pregnancy, geographic location, and race and ethnicity [38].

We also observed that exposure to ambient air pollution during the three months prior to pregnancy and first trimester increased the risk of BDs using multivariate logistic regression analysis. Specifically, exposure to PM_10_, SO_2_ and CO in second and third month of pregnancy were positively associated with BDs. CO exposure in the second month prior to pregnancy was also significantly associated with BDs. These results increased the evidence of a possible correlation between ambient air pollution and BDs among Chinese women. This time frame of susceptibility may be explained by multiple factors. Women of childbearing age develop a new batch of follicles every month, and it takes approximately 85 days for pre-sinus follicles to develop into mature follicles, ready for fertilization. Therefore, three months prior to pregnancy the egg has begun its development and is susceptible to environmental factors [39]. Additionally, the third to eighth week of embryo development is the most sensitive period of development, during which embryonic cells are highly differentiated and susceptible to many teratogenic factors. Considering that the 3 months before pregnancy to the first trimester of pregnancy represent the egg development process and the most sensitive development stage of the fetus, it is likely impacted by air pollution.

This study uniquely examined the effect of exposure to six environmental pollutants on the risk of BDs in minority areas. Our findings are supported by previous published findings, showing a relationship between BDs and PM_10_ and SO_2_ exposure [14–18]. However, the time frame of susceptibility in our study differed from that of previous studies [15, 16]. Different findings may be related to different study methods, different covariate controls, and different exposure levels. We also observed that exposure to CO during the second and third months of pregnancy increased the risk of BDs. In the United States, studies have found a positive association between congenital heart disease and increasing CO exposure during weeks three through eight of pregnancy [40]. Our study expanded the window of observation, allowing temporal associations to be clarified. Zhao et al. also reported that CO exposure levels were associated with the risks of congenital anomalies in 1st trimester [41]. The above studies are consistent with the results of this study, suggesting that atmospheric air pollution before and during pregnancy may increase the risk of BDs. Therefore, environmental agencies have a responsibility to keep working to improve air quality, in order to protect the health of mothers and infants.

Our study had several strengths. All pregnant women in the study range were studied from first 3 months before pregnancy to first trimester of pregnancy, expanding and clarifying the time frame of susceptibility. Additionally, the risk period of birth defects in minority areas was analyzed for the first time. Further, this study included all pregnant outcomes from midwifery institutions and community health services such as live birth, stillbirth and infant death within 7 days, allowing us to analyze births occurring outside the hospital setting. Lastly, this study examined the effect of six kinds of pollutants exposure on the risk of BDs in minority areas for the first time.

Our research has several limitations. First, there were no data on stillbirths and termination of pregnancy before 23 weeks of gestation, which may lead to an underestimation of BDs. We used the air pollution data from air monitoring stations in the same district of the mother’s residence at delivery. Misclassification of exposure is possible if maternal movement and migration occurred during the exposure window of interest in our study. In addition, although several covariates have been tested in this study, we could not rule out other important confounding factors due to missing information.

## Conclusion

This study provided a comprehensive description of ethnic differences in BDs in Southwest China and broadens the evidence of association between air pollution exposure and BDs. Our results indicated an association between exposure to air pollution and BDs. The pollutants PM10, SO2 and CO influenced BDs in the first trimester of pregnancy. SO2 also had an effect on BDs in the second month before the pregnancy. The relationship between birth defects and atmospheric pollutants needs to be further explored in the future. It is important for the government to pay attention to environmental pollution and take early intervention measures to reduce the BDs in order to improve the birth quality in ethnic minority areas.

## Supplementary Information

**Additional file 1: Supplemental Table 1.** Correlation between monthly concentration (ug/m^3^) of pollutants and congenital heart disease.

**Additional file 2: Supplemental Table 2.** Correlation between monthly concentration (ug/m^3^) of pollutants and polydactyly.

**Additional file 3: Supplemental Table 3.** Correlation between monthly concentration (ug/m^3^) of pollutants and cleft lip and/or cleft palate.

## Data Availability

The data that support the findings of this study are available from Management Center of Liuzhou Maternal and Child Health Information, but restrictions apply to the availability of these data, which were used under license for the current study and not publicly available. Data are available from the authors upon reasonable request and with permission from the Management Center of Liuzhou Maternal and Child Health Information.
