# Supplementary material for: The association between ambient air pollution and birth defects in five major ethnic groups in Liuzhou, China
Source: BMC Pediatr. 2021 May 14;21:232. doi: 10.1186/s12887-021-02687-z (PMC8120832; doi:10.1186/s12887-021-02687-z)
Supplement: Supplementary file 1 — Additional file 1: Supplemental Table 1. Correlation between monthly concentration (ug/m3) of pollutants and congenital heart disease. [file 12887_2021_2687_MOESM1_ESM.docx]

| **Supplemental Table 1. Correlation between monthly concentration(ug/m^3^) of pollutants and congenital heart disease** | | | | | | | | | | | |
| --- | --- | --- | --- | --- | --- | --- | --- | --- | --- | --- | --- |
|  | |  |  | | Crude |  |  | Adjusted | |  | |
|  | |  | OR | | 95%CI | P | OR 95%CI P | | | | |
| PM10 | | Before pregnancy | | |  |  |  |  | |  | |
|  | | 1st month | 1.01 | | 0.98-1.08 | 0.88 | 1.03 | 1.02-1.15 | | 0.84 | |
|  | | 2nd month | 1.02 | | 1.00-2.26 | 0.00 | 1.00 | 0.98-1.06 | | 0.78 | |
|  | | 3rd month | 1.28 | | 1.06-3.25 | 0.00 | 1.16 | 1.01-2.24 | | 0.18 | |
|  | | Pregnancy |  | |  |  |  |  | |  | |
|  | | 1st month | 1.05 | | 1.00-1.23 | 0.03 | 1.00 | 0.96-1.42 | | 0.06 | |
|  | | 2nd month | 1.81 | | 1.57-2.12 | 0.01 | **1.32** | **1.23-3.10** | | **0.01** | |
|  | | 3rd month | 1.67 | | 1.42-2.17 | 0.06 | **1.43** | **1.26-2.46** | | **0.02** | |
| SO2 | | Before pregnancy | | |  |  |  |  | |  | |
|  | | 1st month | 1.02 | | 1.00-1.25 | 0.00 | 1.04 | 1.01-1.08 | | 0.62 | |
|  | | 2nd month | 3.17 | | 2.26-4.78 | 0.00 | **1.36** | **1.21-3.44** | | **0.03** | |
|  | | 3rd month | 1.32 | | 1.00-1.56 | 0.00 | 0.98 | 0.91-1.07 | | 0.25 | |
|  | | Pregnancy |  | |  |  |  |  | |  | |
|  | | 1st month | 1.20 | | 1.03-1.36 | 0.01 | 1.12 | 1.06-1.26 | | 0.16 | |
|  | | 2nd month | 0.98 | | 0.97-1.12 | 0.81 | 0.99 | 0.92-1.21 | | 0.32 | |
|  | | 3rd month | 1.97 | | 1.92-3.92 | 0.00 | **1.78** | **1.10-3.64** | | **0.02** | |
| CO | | Before pregnancy | | | |  |  |  |  |  | |
|  | | 1st month ~ | | | 2.62 | 2.13-3.65 | 0.00 | 1.32 | 1.14-1.92 | 0.12 | |
|  | | 2nd month | | | 5.26 | 3.26-6.86 | 0.00 | 1.53 | 1.25-2.34 | 0.10 | |
|  | | 3rd month | | | 2.12 | 1.37-3.13 | 0.00 | 1.06 | 0.87-1.67 | 0.64 | |
|  | | Pregnancy | | |  |  |  |  |  |  | |
|  | | 1st month | | | 1.87 | 1.35-2.76 | 0.00 | 1.30 | 1.03-1.76 | 0.09 | |
|  | | 2nd month | | | 1.16 | 1.17-1.76 | 0.06 | **1.67** | **1.03-2.69** | **0.03** | |
|  | | 3rd month | | | 0.87 | 0.74-1.56 | 0.53 | **1.84** | **1.26-3.73** | **0.02** | |

Abbreviations: OR, odd ratio; CI, confidence interval. Models were adjusted for maternal age, maternal education, birth weight, infant gender, total previous live births, residence and other air pollutants within the same exposure period.
