# Supplementary material for: The association between ambient air pollution and birth defects in five major ethnic groups in Liuzhou, China
Source: BMC Pediatr. 2021 May 14;21:232. doi: 10.1186/s12887-021-02687-z (PMC8120832; doi:10.1186/s12887-021-02687-z)
Supplement: Supplementary file 2 — Additional file 2: Supplemental Table 2. Correlation between monthly concentration (ug/m3) of pollutants and polydactyly. [file 12887_2021_2687_MOESM2_ESM.docx]

| **Supplemental Table 2. Correlation between monthly concentration(ug/m^3^) of pollutants and polydactyly** | | | | | | | | | | | |
| --- | --- | --- | --- | --- | --- | --- | --- | --- | --- | --- | --- |
|  | |  |  | | Crude |  |  | Adjusted | |  | |
|  | |  | OR | | 95%CI | P | OR 95%CI P | | | | |
| PM10 | | Before pregnancy | | |  |  |  |  | |  | |
|  | | 1st month | 0.98 | | 0.92-1.06 | 0.92 | 1.00 | 0.95-1.09 | | 0.79 | |
|  | | 2nd month | 1.05 | | 1.01-2.02 | 0.04 | 1.02 | 0.95-1.06 | | 0.67 | |
|  | | 3rd month | 1.33 | | 1.12-2.98 | 0.00 | 1.14 | 1.02-2.23 | | 0.18 | |
|  | | Pregnancy |  | |  |  |  |  | |  | |
|  | | 1st month | 1.03 | | 0.97-1.32 | 0.02 | 1.02 | 0.96-1.41 | | 0.07 | |
|  | | 2nd month | 1.67 | | 1.58-2.12 | 0.01 | **1.41** | **1.26-3.26** | | **0.01** | |
|  | | 3rd month | 1.68 | | 1.42-2.32 | 0.06 | **1.51** | **1.24-2.76** | | **0.02** | |
| SO2 | | Before pregnancy | | |  |  |  |  | |  | |
|  | | 1st month | 1.05 | | 1.01-1.28 | 0.00 | 1.01 | 1.00-1.19 | | 0.52 | |
|  | | 2nd month | 3.15 | | 2.24-4.16 | 0.00 | **1.53** | **1.18-3.56** | | **0.02** | |
|  | | 3rd month | 1.06 | | 0.98-1.28 | 0.00 | 0.98 | 0.91-1.11 | | 0.34 | |
|  | | Pregnancy |  | |  |  |  |  | |  | |
|  | | 1st month | 1.02 | | 0.98-1.16 | 0.01 | 1.20 | 1.10-1.36 | | 0.15 | |
|  | | 2nd month | 0.92 | | 0.88-1.12 | 0.74 | 0.96 | 0.92-1.24 | | 0.38 | |
|  | | 3rd month | 1.98 | | 2.08-3.96 | 0.00 | **1.78** | **1.16-3.65** | | **0.02** | |
| CO | | Before pregnancy | | | |  |  |  |  |  | |
|  | | 1st month ~ | | | 2.36 | 2.13-3.68 | 0.00 | 1.35 | 1.03-1.95 | 0.12 | |
|  | | 2nd month | | | 4.24 | 3.38-7.67 | 0.00 | 1.68 | 0.96-2.32 | 0.10 | |
|  | | 3rd month | | | 2.16 | 1.66-2.84 | 0.00 | 1.02 | 0.87-1.73 | 0.65 | |
|  | | Pregnancy | | |  |  |  |  |  |  | |
|  | | 1st month | | | 1.87 | 1.39-2.58 | 0.00 | 1.26 | 0.98-1.83 | 0.16 | |
|  | | 2nd month | | | 1.19 | 0.98-1.76 | 0.07 | **1.74** | **1.06-2.86** | **0.03** | |
|  | | 3rd month | | | 0.98 | 0.82-1.32 | 0.64 | **1.86** | **1.43-3.86** | **0.02** | |

Abbreviations: OR, odd ratio; CI, confidence interval. Models were adjusted for maternal age, maternal education, birth weight, infant gender, total previous live births, residence and other air pollutants within the same exposure period.
