# Supplementary material for: The association between ambient air pollution and birth defects in five major ethnic groups in Liuzhou, China
Source: BMC Pediatr. 2021 May 14;21:232. doi: 10.1186/s12887-021-02687-z (PMC8120832; doi:10.1186/s12887-021-02687-z)
Supplement: Supplementary file 3 — Additional file 3: Supplemental Table 3. Correlation between monthly concentration (ug/m3) of pollutants and cleft lip and/or cleft palate. [file 12887_2021_2687_MOESM3_ESM.docx]

| **Supplemental Table 3. Correlation between monthly concentration(ug/m^3^) of pollutants and cleft lip and/or cleft palate** | | | | | | | | | | | |
| --- | --- | --- | --- | --- | --- | --- | --- | --- | --- | --- | --- |
|  | |  |  | | Crude |  |  | Adjusted | |  | |
|  | |  | OR | | 95%CI | P | OR 95%CI P | | | | |
| PM10 | | Before pregnancy | | |  |  |  |  | |  | |
|  | | 1st month | 0.96 | | 0.89-1.16 | 0.90 | 0.97 | 0.95-1.06 | | 0.98 | |
|  | | 2nd month | 1.06 | | 1.01-2.17 | 0.04 | 1.02 | 0.96-1.08 | | 0.64 | |
|  | | 3rd month | 1.22 | | 1.09-2.76 | 0.00 | 1.04 | 1.02-2.34 | | 0.12 | |
|  | | Pregnancy |  | |  |  |  |  | |  | |
|  | | 1st month | 1.05 | | 0.94-1.63 | 0.02 | 1.01 | 0.99-1.54 | | 0.06 | |
|  | | 2nd month | 1.83 | | 1.54-2.25 | 0.01 | **1.32** | **1.17-3.62** | | **0.01** | |
|  | | 3rd month | 1.64 | | 1.31-2.47 | 0.05 | **1.47** | **1.14-2.82** | | **0.02** | |
| SO2 | | Before pregnancy | | |  |  |  |  | |  | |
|  | | 1st month | 1.03 | | 0.97-1.46 | 0.00 | 1.12 | 1.01-1.24 | | 0.47 | |
|  | | 2nd month | 3.36 | | 2.17-4.08 | 0.00 | **1.62** | **1.21-3.46** | | **0.02** | |
|  | | 3rd month | 1.04 | | 0.96-1.62 | 0.00 | 0.96 | 0.90-1.21 | | 0.28 | |
|  | | Pregnancy |  | |  |  |  |  | |  | |
|  | | 1st month | 1.12 | | 0.99-1.26 | 0.01 | 1.10 | 1.09-1.46 | | 0.12 | |
|  | | 2nd month | 0.98 | | 0.86-1.22 | 0.56 | 0.95 | 0.90-1.44 | | 0.45 | |
|  | | 3rd month | 1.97 | | 2.15-3.87 | 0.00 | **1.69** | **1.16-3.84** | | **0.02** | |
| CO | | Before pregnancy | | | |  |  |  |  |  | |
|  | | 1st month ~ | | | 2.27 | 2.36-3.88 | 0.00 | 1.25 | 1.02-1.90 | 0.15 | |
|  | | 2nd month | | | 4.14 | 3.27-5.66 | 0.00 | 1.08 | 0.92-2.12 | 0.12 | |
|  | | 3rd month | | | 2.25 | 1.68-2.74 | 0.00 | 1.12 | 0.94-1.82 | 0.59 | |
|  | | Pregnancy | | |  |  |  |  |  |  | |
|  | | 1st month | | | 1.74 | 1.57-2.65 | 0.00 | 1.41 | 0.98-2.13 | 0.07 | |
|  | | 2nd month | | | 1.37 | 0.97-1.89 | 0.07 | **1.83** | **1.16-2.97** | **0.03** | |
|  | | 3rd month | | | 1.05 | 0.93-1.46 | 0.58 | **1.95** | **1.28-3.92** | **0.02** | |

Abbreviations: OR, odd ratio; CI, confidence interval. Models were adjusted for maternal age, maternal education, birth weight, infant gender, total previous live births, residence and other air pollutants within the same exposure period.
